# Efficacy of personalised text message intervention in reducing smoking frequency and amount for non-abstinent smokers: A double-blind, randomised controlled trial

**DOI:** 10.7189/jogh.13.04133

**Published:** 2023-10-27

**Authors:** Haoxiang Lin, Min Li, Li Xiao, Chun Chang, Gordon G Liu

**Affiliations:** 1 Institute for Global Health and Development, Peking University, Beijing, China; 2 DeZhou Center for Disease Control and Prevention, Beijing, China; 3 China Center for Health Education, Beijing, China; 4 School of Public Health, Peking University, Beijing, China

## Abstract

**Background:**

Emerging evidence supports the efficacy of mobile phone interventions for smoking cessation. However, behaviour changes of smokers who fail to reach abstinence and the related psychological mechanism are still understudied. We aimed to evaluate the efficacy of a behaviour change theory-based smoking cessation intervention delivered through personalised text messages from the perspective of smokers who fail to reach abstinence.

**Methods:**

We conducted a two-arm, double-blind, randomised controlled trial, with the intervention group receiving personalised text messages developed specifically for this study, and the control group receiving non-personalised ones related to smoking cessation. These messages were sent over a period of three months. We looked at three outcomes: changes in smoking frequency, in smoking amount, and in protection motivation theory (PMT) construct scores.

**Results:**

We obtained smoking cessation results for 722 participants who went through the randomisation process (intervention: 360, control: 362; block randomisation design). Overall, 32.3% of baseline daily smokers in the intervention group and 20.0% in the control group changed to weekly smokers during the follow-up period (*P* < 0.001), while 43.4% of consistent daily smokers in the intervention group and 32.8% in the control group continuously reduced their smoking amount (*P* < 0.001). We observed associations between the intrinsic rewards of smoking and changes from daily to weekly smoking, the perceived severity of smoking and reductions in smoking amount, as well as the self-efficacy of quitting and changes from daily to weekly smoking/reductions in smoking amount.

**Conclusions:**

We found that a personalised text message-based intervention was more likely to decrease the frequency and amount of smoking in smokers who failed to reach abstinence compared to a non-personalised one. We also explored the possible psychological mechanism of such positive effects. Here we provide evidence for countries to consider the promotion of smoking cessations using behaviour theory-driven personalised text messages, which may be more cost-effective than traditional approaches.

**Registration:**

Peking University: ChiCTR2100041942.

Studies have shown that mobile cessation programs can encourage smokers to quit smoking and are affordable even in resource-poor settings, but have mostly focused on showing the framework of an intervention and reporting how many smokers reach abstinence (normally the three- or six-month sustained abstinence rate) [1-3]. Other components, i.e. the behaviour change of smokers who fail to reach abstinence and the related psychological mechanism, are still understudied.

As even the most effective treatments help just one in four smokers quit in the long-term, and as mobile cessation may have a much lower quitting rate than pharmacotherapy, exclusively measuring the quitting rate is insufficient [4]. Therefore, we believe it necessary to investigate the change in smokers who fail to reach abstinence and the psychological determinants of positive change in order to comprehensively evaluate mobile cessation efficacy and provide insight into how to use minimal interventions to achieve maximum effects.

Until recently, the psychological mechanism of promoting quitting behaviour using mobile health interventions have been explored by only a few studies [5,6], with most finding some psychological determinants of abstinence. For example, a personalised text message program found that the intrinsic reward of smoking and self-efficacy of quitting were significantly associated with quitting [5]. However, the positive behavioural changes and determinants of success at different reduction stages of smokers who fail to reach abstinence remain under-researched. One dimension of such behavioural changes is the continuous reduction in smoking amount, which is especially valuable in less intensive smoking cessation interventions, such as text message-based ones

Individuals are usually advised to quit smoking abruptly on a designated “quit” day [7,8]; however, a Cochrane review published in 2019 found that reducing smoking behaviour prior to quitting could also be viable [9]. Therefore, we believe that a stable reduction in the number of cigarettes post-intervention could be a meaningful indicator for measuring the effects of a mobile cessation intervention, particularly for smokers who fail to reach abstinence.

Reducing smoking frequency can also be a pathway of a positive change for smokers, as studies support the value of lengthening the time between cigarettes [9-11]. First, a shift form daily to weekly smoking reduces the average daily intake of nicotine, and consequently, decreases nicotine dependence. Second, a reduction in smoking frequency represents a step towards cessation, thereby increasing self-efficacy and the likelihood of achieving abstinence, while also providing a more achievable goal compared to complete cessation for some heavy smokers who use mobile cessation support. It may also help with participant retention and provide an opportunity to increase an individual’s motivation to quit.

Studies have found that personalised and behaviour change theory-based interventions could be more effective than only providing broad, non-specific advice [12,13] because self-relevant information can easily engage attention, increase motivation for information processing, and cause more behavioural changes [14]. Most personalised communications are achieved through ongoing evaluation; allowing smokers to join the information generation process could also increase compliance. We believe that reducing smoking frequency and amount are two important indicators of abstinence. A survey in the UK found that 40% of quitting attempts involved an initial cut-down in consumption [9], while data from the USA showed that nearly half of smokers planning to quit would choose reduction over abrupt cessation [15]. We thus hypothesised that a personalised text message-based intervention would more likely lead to reductions in smoking amount and frequency in smokers who fail to reach abstinence than a non-personalised one.

We previously conducted a randomised controlled trial (RCT) to address the evidence gap and comprehensively evaluate the impact of mobile cessation interventions. We found that a behaviour change theory-based smoking cessation intervention through personalised text messages was more effective in reducing the biochemically verified six-month sustained abstinence rate than one based on non-personalised messages (6.9% vs 3.0% (adjusted odds ratio (OR) = 2.66; 95% confidence interval (CI) = 1.21-5.83)) [3].

Here we aimed to evaluate the efficacy of a behaviour change theory-based smoking cessation intervention delivered through personalised text messages from the perspective of smokers who fail to reach abstinence. We focused on behaviour change, such as reducing smoking amount or frequency, of participants who continued smoking during the follow-up period. We also further sought to explore the psychological mechanism of positive change.

## METHODS

### Study design and participants

We conducted two-arm, double-blind, randomised controlled trial in five cities in China (Beijing, Dezhou, Baotou, Yakeshi, and Linzi), with participants randomised to an intervention or control group between April 2021 and July 2021.

We included daily or weekly smokers aged ≥18 who owned a mobile phone and used WeChat, and who agreed to participate by signing an informed consent form. We excluded individuals who had received any smoking cessation treatment within 30 days or had a diagnosis of any mental illnesses (either current or past).

The Peking University registered the clinical trial (ChiCTR2100041942). We reported this study the following CONSORT reporting guidelines for randomised studies [16].

### Development of the intervention framework

We based the intervention’s theoretical framework on the transtheoretical model (TTM) and protection motivation theory (PMT). For the TTM, we used several strategies to strengthen behaviour changes and/or to achieve the next stage. On the pre-quit date, we sent messages related to consciousness raising, dramatic relief, and environmental re-evaluation to smokers with weak quitting intentions, and those related to stimulus control, self-liberation, and reinforcement management to smokers with strong quitting intentions. After the quit date, we sent messages on consciousness raising, dramatic relief, environmental re-evaluation and self-re-evaluation to smokers who had relapsed.

All the participants also received messages by PMT related to the severity and susceptibility of smoking, intrinsic and extrinsic rewards of smoking, self-efficacy and response efficacy and response cost of quitting. The motivational messages consisted of 14 subgroups with a total of 400 text messages. A more detailed framework of the text and intervention based on the TTM and PMT is reported separately [3,5,6]. The example of the intervention messages are presented in Table S1 in the **Online Supplementary Document**.

### Intervention tool: WeChat

WeChat is the most popular Chinese social media app, which our information technology team used to deploy the messages. The application evaluated the PMT construct score by asking questions and recording information; then, it automatically calculated the lower score of the sub-content that needed to be strengthened. Specifically, the scale comprised 21 items using a seven-point Likert-type scale with responses ranging from 1 (definitely disagree) to 7 (definitely agree). Each construct subscale of PMT included three items, and we computed the mean of the subscale score. The TTM stage was also identified according to the smoking status of users. Then, WeChat issued personalised text messages according to the evaluation, as detailed elsewhere. Details on the scales and the processes are available elsewhere [11,12,17,18]. The WeChat application determined each smoker’s quitting intention through a five-point scale ranging from 1 (not at all likely) to 5 (very likely) to in the likelihood that they would try to quit in the next six months [13].

### Recruitment

We advertised the trial to smokers through paper advertising (leaflets), digital advertising (WeChat) and staff (teachers and leaders). Potential participants contacted the local Disease Control and Prevention Center to register their interest. The research assistants explained the study to each potential participant and preliminarily assessed his or her eligibility. All eligible smokers were told they needed to come to a place on a fixed date to finalize the recruitment process.

### Randomisation and blinding

Participants were required to register through WeChat following recruitment. We used a randomised block design, with the participant score on the Fagerström Test for Nicotine Dependence (range = 0-10, with higher scores indicating greater dependence) treated as a stratified factor [19]. The app automatically generated two blocks based on the test score, with block 1 comprising participants with low or moderate nicotine dependence (score of 0-6 points) and block 2 comprising those with high nicotine dependence (score of 7 points). Eligible participants were assigned to the intervention group or the control group within each block by simple randomisation. The WeChat system was also used to balance demographic characteristics. Randomisation was fully computerised and automated with equal allocation. The researchers and participants were all blinded. Our randomisation technique has been detailed elsewhere [3].

### Intervention

Participants allocated to the intervention group received the intervention program consisting of one to two personalized text messages a day for three months.

Control group participants received non-personalised text message developed by the National Cancer Institute (NCI); this intervention was based on well-established cognitive-behavioural cessation approaches and contained 91 messages. Participants received approximately one message a day for three months. The text messages provided encouragement, practical advice to help maintain cessation, and information on the health effects of smoking. The details of the control group can be found elsewhere [14].

### Assessment

All participants in the two groups were instructed to attend face-to-face follow-ups with research staff at one, three, and six months after randomisation. The outcomes were as follows:

1. Frequency reduction – achieved when a daily smoker (at baseline) had not successfully quit according to the evaluation of biochemically verified six-month sustained abstinence rate, but had reduced the smoking frequency to weekly smoking and maintained that smoking frequency during the follow-up period.
2. Smoking amount reduction – achieved when a daily smoker (at baseline) had not successfully quit according to the evaluation of biochemically verified six-month sustained abstinence rate, but had continuously reduced the smoking amount or reduced to a lower level and maintained the lower level during the follow-up period.
3. Changes in PMT construct scores, including perceived severity, perceived vulnerability, intrinsic rewards of smoking, extrinsic rewards of smoking, self-efficacy of quitting, response efficacy of quitting, and response cost of quitting.

### Statistical analysis

We based the sample size calculation on the formula for a two-arm RCT. To achieve 80% power with a significance level of 0.05 (two sided), we required a sample size of 280 individuals in each group. Assuming 20% attrition in the follow-up measurements, we set the required sample size at 672, as reported elsewhere [3].

We used descriptive statistics to compare the baseline status between the two groups, after which we conducted an intention-to-treat (ITT) analysis and the Russell Standard (RS) for each group. All randomised subjects were included in the denominator for calculating abstinence rates, except for unavoidable loss to follow-up (died or moved to an untraceable address). Those who declined to be involved in subsequent data collection were counted as smokers [20].

To evaluate the impact on reducing the frequency of smoking (the amount of smoking), we first excluded non-daily smokers at baseline and participants who reached biochemically verified six-month sustained abstinence. Then, we identified participants who changed smoking behaviour from daily to weekly and remained weekly smokers (who had continuously reduced the smoking amount or reduced to a lower level and maintained the lower level) until all the follow-up visits were completed and categorised them as “changers”. We used descriptive statistics and χ^2^ tests to compare differences in the percentage of “changers” between the two groups. For PMT construct scores, we compared baseline and each follow-up time point difference between the two groups by t-*t*ests.

Finally, we used generalised estimating equations (GEEs) to estimate the psychological determinants of reducing the frequency of smoking and reducing the smoking amount to determine why the intervention group had a more positive change. In the first set of models, we set a dummy variable indicating whether the respondent reduced the frequency of smoking compared to the last follow-up visit (if yes = 1, otherwise = 0) as the dependent and the PMT construct scores as the independent variable, controlling for education level, living area, nicotine dependence, and monthly income. We chose an autocorrelation structure for the matrix structure and logistic regression for the model setting. In the second set of models, we changed the dependent variable to whether the respondent reduced their smoking amount compared to the last follow-up visit (if yes = 1, otherwise = 0). The results are presented as ORs and 95% CIs. We performed all analyses in SPSS, version 11.0 (SPSS, Chicago, Illinois, USA).

### Ethics considerations

The Ethics Committee of Peking University Health Science Center (IRB00001052-30063) reviewed and approved the trial. All the participants signed informed consent forms before randomisation and knew they could withdraw from the study at any time. All patient information was accessible only by personnel participating in the study. We did not provide money to participants, but provided gifts (towel, umbrella, or cup) if they completed one follow-up visit.

### Patient and public involvement

Patients or the public were not involved in the design, conduct, reporting, or dissemination plans of our research.

## RESULTS

We screened 780 smokers and randomly assigned 722 (92.6%) to the intervention or control group, all of whom were Chinese. Both groups were well-balanced in their baseline characteristics Figure S1 and Table S2 in the **Online Supplementary Document**). We excluded 43 potential participants who were not eligible.

**Figure 1:**
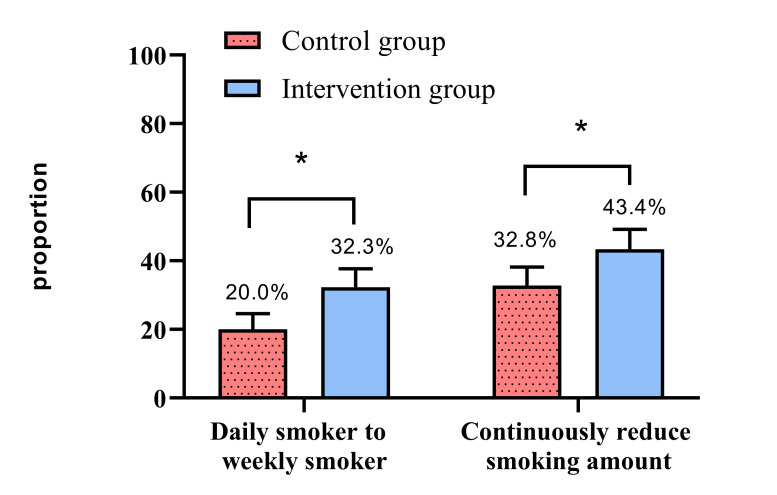
Changing smoking behaviour and reducing smoking amount between the two groups of current smokers. *P < 0.001.

We found that 32.3% (n = 93) of baseline daily smokers in the intervention and 20.0% (n = 60) in the control group changed to weekly smokers during the follow-up period (*P* < 0.001), while 43.4% (n = 125) of consistent daily smokers in the intervention and 32.8% (n = 95) in the control group continuously reduced their smoking amount (*P* < 0.001) (**Figure 1**).

**Figure 2:**
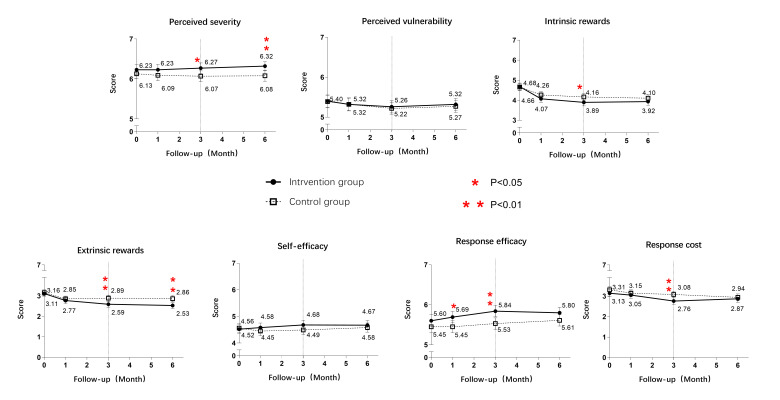
The point-to-point comparison of protection motivation theory construct scores.

We found no statistically significant differences in any of the seven PMT construct scores between the two groups at baseline (**Figure 2**). However, as the intervention began, the intervention group had higher scores for perceived severity of smoking and response efficacy of quitting and lower scores for intrinsic and extrinsic rewards of smoking and response cost of quitting.

**Figure 3:**
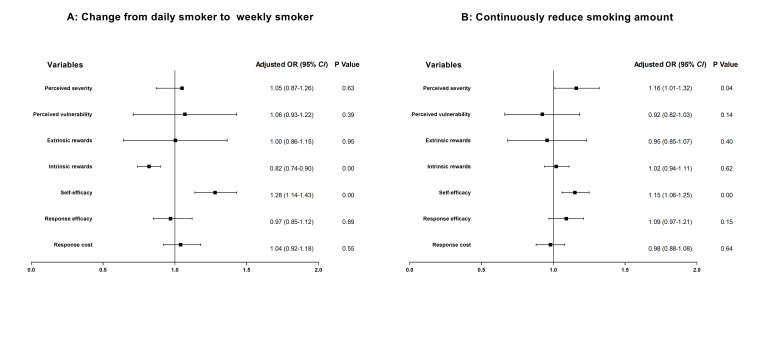
Generalised estimating equations for changing smoking behaviour and reducing smoking amount on protection motivation theory subconstructs. We adjusted the analysis for living area, educational level, monthly income, and nicotine dependence at baseline.

In the last GEE regression (**Figure 3**), we estimate the psychological determinants of behaviour change. We found associations between the intrinsic rewards of smoking (OR = 0.82; 95% CI = 0.74-0.90) and changes from daily to weekly smoking, the perceived severity of smoking (OR = 1.16; 95% CI = 1.01- 1.32) and reductions in smoking amount, as well as self-efficacy of quitting and changes from daily to weekly smoking (OR = 1.28; 95% CI = 1.14- 1.43) as well as reductions the smoking amount (OR = 1.15; 95% CI = 1.06-1.25).

## DISCUSSION

To our knowledge, this is the first randomised controlled trial using personalised text messages for a mobile cessation intervention with a positive control group design conducted in a a country with limited tobacco control policy, and the first to report the effects from the perspective of smokers who failed to reach abstinence. Considering the high response rate from participants, our findings provide a deep insight into the positive change resulting from a text message-based smoking cessation intervention in the context of China.

We found personalised text messages encouraged more smokers to reduce their smoking amount and frequency. Because most mobile cessation interventions have a relatively lower abstinence rate (<10%) [1,21], an intervention which can bring positive change to another 90% of participants is highly relevant. Moreover, reducing smoking behaviour and stabilising at a lower level means that a stronger quitting intention has been developed, which may result in higher quitting rates in the future.

However, abrupt quitting interventions are generally recommended in clinical treatment guidelines and suggested by medical professionals. However, a Cochrane review found no difference in outcomes between reducing smoking over time and quitting abruptly; thus, individuals could be given a choice of how to quit [9]. We do not intend to evaluate or challenge any smoking cessation guidelines, but we believe that reducing one’s smoking frequency or amount should be treated as a positive change for smokers.

We also managed to identify the psychological determinants of reducing smoking frequency from daily to weekly and reducing smoking amount. The comparison between groups showed that the personalised intervention decreased the intrinsic rewards of smoking and increased perceived severity of smoking, making it a notable determinant of positive change in smoking behaviour. It also provides some indications as to why a personalised intervention is more likely than a non-personalised intervention to encourage smokers who fail to reach abstinence to reduce their smoking amount and frequency.

This study has several limitations. First, most of our participants were male and Chinese, limiting generalisability to other populations. Second, our follow-up period only lasted until six months after the intervention was implemented, and a long-term evaluation may produce different results. Third, only daily regular smokers reported their smoking amount at baseline and each follow-up point. Therefore, we only included participants who were daily smokers at baseline when measuring the effect on smoking reduction and frequency-changing results. Fourth, the results can be biased because we only conducted point-to-point comparisons for PMT construct scores; more robust analyses should account for the correlated nature of the repeated measures in the data. Fifth, the results can also be influenced by other important issues, such as recall or selection bias and social acceptability problems, since the data were collected from individuals who decided to participate in our study.

However, our study has important implications. Although research has found that text message-based interventions could provide smoking cessation support, their low quitting rate remains a challenge for expanding their usage to promote smoking cessation and gaining government support. Our study suggest that text message-based interventions can both help smokers quit and bring change those who fail to reach abstinence, making it suitable for countries with limited smoking cessation resources by allowing minimal expenses for maximum effects. We also found some psychological determinants of smoking reduction; among them, self-efficacy of quitting was associated with reductions in both smoking frequency and amount. This finding was also confirmed by other smoking cessation studies showing that self-efficacy was the most significant variable associated with forwards progress towards quitting [5,6]. Those identified psychological determinants could help with further development of specific intervention measures for different groups of people.

## CONCLUSIONS

Based on a six-month evaluation, we found that a personalised text message-based intervention was more likely to decrease the frequency and amount of smoking in smokers who failed to reach abstinence compared to a non-personalised one. Further GEE analyses suggested the possible psychological mechanism of such positive effects. Our findings could help other countries that are starting to design or promote text message-based smoking cessation interventions.

## Additional material


Online Supplementary Document
