# Supplementary material for: Efficacy of personalised text message intervention in reducing smoking frequency and amount for non-abstinent smokers: A double-blind, randomised controlled trial
Source: J Glob Health. 2023 Oct 27;13:04133. doi: 10.7189/jogh.13.04133 (PMC10602206; doi:10.7189/jogh.13.04133)

**Supplement Table 1. The example of intervention messages**

| | **Example text message** |
|---|---|
| Increase severity and susceptibility | 1.Half of the smokers will die early from smoking<br>2.Compared with smoking regular cigarettes, smoking "low-tar cigarettes" does not reduce the harm of smoking. |
| Decrease response cost, intrinsic and extrinsic rewards | 1.Quitting smoking is actually the most cost-effective health care measure<br>2.Currently, everyone is pursuing a healthy lifestyle; smoking is no longer a social trend. |
| Increase self-efficacy and response efficacy | 1.If there is a smoker in your home, you can encourage him or her to quit smoking together with you.<br>2.Your efforts are for a better life; many people like you are quitting smoking with us now |

Supplement Figure 1. Study flowchart

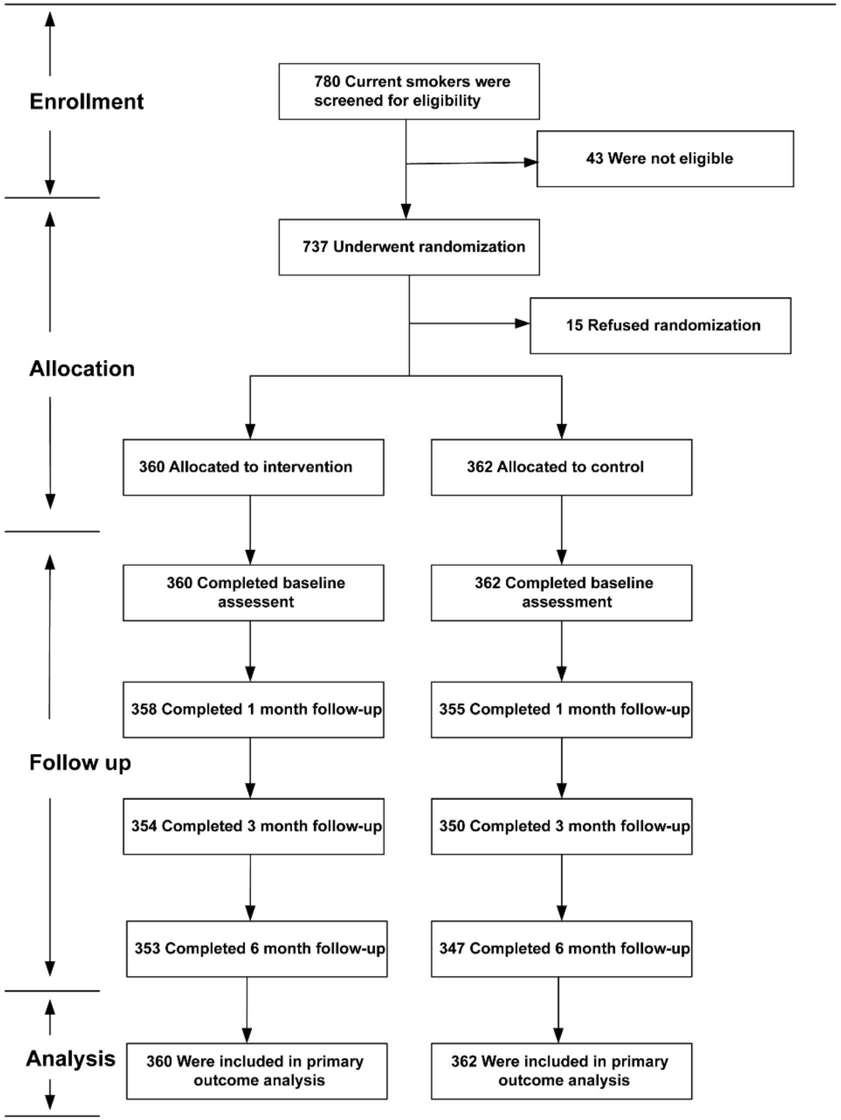

Supplement: Online Supplementary Document [file jogh-13-04133-s001.pdf]
